# Revisiting Partial Hepatectomy of Large Hepatocellular Carcinoma in Older Patients

**DOI:** 10.1038/s41598-018-32798-0

**Published:** 2018-09-28

**Authors:** Guoyong Chen, Jiabin Zhang, Jianjun Sun, Sidong Wei, Jianbin Chen, Hui Ren, Shaotang Zhou

**Affiliations:** 1 Section 5 of Hepatopancreaticobiliary Surgery, Henan Provincial People’s Hospital, Zhengzhou, Henan 450003 China; 2 Center of Hepatopancreaticobiliary Surgery and liver transplantation, 302 Hospital, Beijing, 100039 China

## Abstract

Hepatectomy of large hepatocellular carcinomas (>10 cm) in over 70 year-old patients is presumed futile. We retrospectively reviewed 5970 patients with liver tumors Jan 2010 through Dec 2016 in our institute, of them, 37 older patients with large hepatocellular carcinomas staged I-III and Child-Pugh A liver functions receiving conservative treatments (conservative group, n = 37) and 16 older patients with large hepatocellular carcinomas staged I- III who underwent partial hepatectomy (resection group, n = 16) were included, the risk factors for poor survival were analyzed by univariate and multivariate analyses. Compared with the conservative treatments, Partial hepatectomy achieved better median survival time (25.5 months versus 11 months, log-rank = 0.0001) and better median performance status (1 versus 3, *p* = 0.023), there was different in Charlson comorbidity index (*p* = 0.019). For the conservative group, the 3-month, 1, 2, 3-year survival rate was 78.4%, 43.2%, 5.4%, 0%; for the resection group, The 3-month, 1, 2, 3-year survival rate was 100%, 93.7.2%, 56.3%, 12.5%; Multivariate Cox regression analysis showed the Charlson comorbidity index and the performance status associated with poor outcomes of those patients (*p* = 0.001, 0.018, respectively). Resections of large hepatocellular carcinomas in older patients can be performed safely to prolong life expectancy and improve life quality with or without cancer recurrence.

## Introduction

The incidence of liver cancer increases by approximately 700,000 patients worldwide each year, and over half of the global incidence and mortality of liver cancer occurs in China^1^. Hepatocellular carcinoma (HCC) represents more than 90% of primary liver cancers and is closely related to viral infections, especially in Eastern Asian. With the global aging of population and asymptomatic progression of liver tumors, large hepatic tumors are likely to occur in the older population. In Japan, the median age at the time of liver cancer diagnosis is 63 years in men and 69 years in women^2^. In China, more people are undergoing physical examinations in the current aging-friendly era, and more liver tumors are being detected. With the advances in liver surgery and medical supportive care, partial hepatectomy has received wide acceptance as a first-line treatment modality for liver tumors. The surgical outcomes for large hepatic tumors vary among different liver centers. Several studies have shown that the 5-year survival rate is unfavorably low^3,4^. Ronald *et al*. reported that about 33% of patients with a large HCC enjoyed life for 5 years^5,6^, similar to those with a smaller HCC. Age or size per se is not the contraindication for liver resections, and hepatectomy has been validated as an effective and safe technique in the older population^7–9^. However, large tumors in patients of advanced age are a challenge for surgeons in terms of planning curative treatment because of shorter lifetime of these older patients, the higher risk of major complications, limited treatment options and the high recurrence tendency^10,11^. Significant comorbidities such as cardiovascular disease, chronic obstructive pulmonary disease, and renal dysfunction can pose great problems for patients undergoing aggressive surgical treatment for liver tumors, especially large tumors, and the combination of a large liver tumor and advanced age surely precludes liver transplantation and percutaneous ablation. Large tumors develop insidiously and devastate the patient’s health asymptomatically. After a tumor has been detected, a patient and his or her family often develop oncophobia over time and request that tumors preferably be removed. In the absence of curative treatment options, cancer-related distress or a wait-to-die intention may result in tragedies including suicide because tumors are frequently associated with dread and death for many patients. Hence, the treatment of an older patient with a large HCC is a pending issue for surgeons. We offer surgery to the selected patients who maintain Child–Pugh class A liver functions and potentially carry a better survival benefit after undergoing major operations. The present study is the first to report the outcomes of partial hepatectomy versus conservative treatment in older patients with large hepatic tumors.

## Methods and Patients

**Figure 1:**
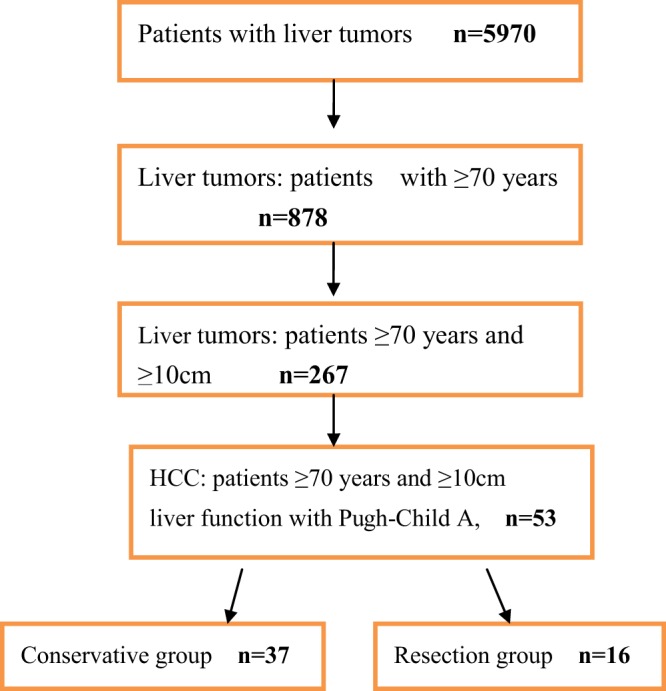
Workflow of all patients with liver tumors shortlisted into two groups.

In the present study, patients aged ≥70 years were considered older or advanced age. The medical records of older patients with large liver tumors from our institution were reviewed from January 2010 through December 2016. The records of 5970 patients with liver tumors in our institute were reviewed; 878 patients were aged ≥70 years. The number of patients aged over 70 year with large HCC was 267, benign or metastatic tumors were excluded, and data on 53 older patients with large tumors who carried Child–Pugh class A liver functions was teased out and divided into the conservative group (n = 37) and the resection group (n = 16) (Fig. 1), patients’ preoperative status, survival, cancer recurrence, Eastern Cooperative Oncology Group (ECOG) performance status, Charlson comorbidity index (CCI)^12^, TNM staging and other parameters were collected for two groups (Table 1). This study was conducted in accordance with the Declaration of Helsinki and approved by the ethics committees of Henan Provincial People’s Hospital and 302 Hospital. Written informed consent was obtained from all patients.

**Table 1:** Characteristic for conservative group and resection one.

	Conservative group (n = 37)	Resection group (n = 16)	*p*
Sex (M/F)	32/5	14/2	0.328
Age	74.1 ± 4.2	74.7 ± 5.1	0.248
Original diseases			0.133
HBV	19	12	
HCV	6	2	
mild Cirrhosis	17	7	
Comorbidities			0.077
Hypertension	19	2	
Cardiac	14	5	
Diabetes	5	3	
CCI*	8.7 ± 1.7	7.6 ± 0.8	0.019
AFP (ng/ml)	1209 (6–34555)	21.5 (2.9–3000)	0.955
Child-Pugh score	6	5	0.802
TBil (µmol/l)	34.8 ± 4.2	25.2 ± 4.7	0.213
HGB (mg/dl)	11.5 ± 1.74	12.1 ± 1.54	0.411
PLT (×10^9^/l)	124.3 ± 8.5	130.1 ± 12.2	0.605
Tumor size	12.1 (10.1–17)	11.3 (10.3–16)	0.687
TNM stage			0.053
I	15	5	
II	11	8	
IIIA	5	2	
IIIB	2	1	
IIIC	2	0	
ECOG	3	1	0.023
Median Survival (months)	11	25.5	0.0001

The data of mean ± standard deviation was presented for measurement variables, median (range) or number for categorical variables. CCI*: Charlson comorbidity index.

### Conservative group

Of the above-described patients, 37 older patients with large liver tumors and Child–Pugh class A liver functions comprised the control group, they received conservative treatments including supportive cares, transcatheter arterial chemoembolization (TACE), radiotherapy, and/or herbal medications. Five patients underwent 1 TACE session, 6 patients underwent 2 TACE sessions, 1 patient underwent 4 TACE sessions, 1 patient underwent 17 TACE sessions, 2 patients underwent gamma radiotherapy, 3 patients underwent 3 argon-helium knife cryosurgery procedures, and the remaining 21 patients received herbal medications and/or symptomatic treatments only.

### Resection group

In January 2010, we commenced radical treatments for older patients with large liver tumors; 16 patients who underwent this curative therapy were included in the present study through Dec 2016. The indication was a liver tumor measuring >10 cm in diameter, Child–Pugh class A liver function and no surgery-resistant comorbidities (Table 2).

**Table 2:** Pre-operational characteristics of resection group. BMI: body mass index; HP: blood hypertension; DM: Diabetes Mellitus; HBV: hepatitis B Virus; HCV: hepatitis C Virus; LVDD: Left ventricular diastolic dysfunction. Cirrhosis*: Cirrhosis was mild.

Case	Age/sex	BMI (kg/m^2^)	Comorbid	Viral	AFP	Size (cm)	TNM staging
1	74/M	27.1	HP, Stroke	no	9.8	11.4	IIIa
2	70/F	28.3	Cirrhosis	no	279.3	11	I
3	70/M	22.4	LVDD	no	76.8	11.6	I
4	87/M	27.7	Cirrhosis*	HBV	4.37	12	IIIb
5	70/M	27.5	DM, HP	HBV	49.9	13	IIIa
6	81/M	18.5	LVDD, Stroke	HBV	309.4	11.5	II
7	73/M	19.7	Cirrhosis	HBV	6.7	11	II
8	77/M	17.8	no	no	19.7	11.5	I
9	74/M	21.4	LVDD	HBV	235.8	11	I
10	70/M	27.1	DM, Cirrhosis	HBV	716	16	II
11	70/F	22.1	DM, HP	HBV	197.2	10.5	II
12	74/M	25.5	HP, Cirrhosis	HBV	319.9	10.6	II
13	70/M	23.1	Cirrhosis	HCV	137.1	11.6	II
14	76/M	27.1	Cirrhosis	HCV	240.4	10.3	II
15	74/M	22.9	Cirrhosis	HBV	100.3	13.4	II
16	73/M	29	Cirrhosis	HBV	28.3	10.6	I

Partial hepatectomy was performed by a senior surgeon to eliminate or reduce the tumor burden for extending the patient’s lifetime and providing tumor-related relief. When patients and their immediate family strongly requested radical resection, written informed consent was obtained, including detailed information on the benefits of hepatectomy, potential major complications and the risk of cancer recurrence.

Partial hepatectomy was scheduled after evaluation of the patient’s operative tolerance. We comprehensively evaluated the liver, kidney, lung, and heart function to ensure that the patient had Child–Pugh class A or corrected class A liver functions and that an adequate remnant volume of liver parenchyma was fully analyzed prior to the resection. In patients with splenomegaly or hypersplenism, measurement of the platelet count and an endoscopic esophagogastric examination were scheduled to determine the degree of esophageal varices; routine preoperative imaging studies included abdominal computed tomography and magnetic resonance imaging. Intraoperative ultrasound was used to locate additional tumors and the main hepatic vessels. Tumor thrombi in the main portal vein or inferior vena cava or cancer invasion of the diaphragm were not absolute contraindications^9^. Numerous or distant metastases in the peritoneal or thoracic cavity were considered unresectable (Staged IV). A low central venous pressure was applied during anesthesia before parenchymal transection. Precision hepatectomy was preferred to maintain a sufficient amount of parenchyma^13^. After initial assessment of the resectability by exploration, the inflow blood supply to the lobes or segments to be resected was divided for selective occlusion. Parenchymal transection was performed using the clamp-crushing technique, which expedites the transection process. The intermittent Pringle maneuver with 15- and 5-min release periods was used. No total vascular exclusion technique was applied in the resection group. Hemostasis during parenchymal transection was achieved with a monopolar electrotome and fine suturing. Blood transfusion was started when the hemoglobin level decreased to <90 g/L. Top priority was given to preservation of the remnant liver function by expediting transection. Coagulant agents were not routinely used to enhance hemostasis. Before the operation, a dose of potent antibiotic was conventionally prescribed for prophylaxis against infection and one more dose was added when hepatectomy lasted over 4 hours, and ultrasound was used to monitor the liquid collection or thrombosis in the hepatic vessels. A drainage tube was maintained for 1 week to monitor and collect fluid or bile leakage from the cut surface. After hepatectomy antibiotics was administered daily until stable liver functions.

Postoperative morbidity was categorized into minor and major complications following the Classification of Surgical Complications by Clavien, *et al*.^14^.

### Follow-up

For the conservative group, information on the patients’ ECOG performance status was obtained from the medical records or by telephone until death^15^. We set 2 years when an over 70 year old patient with large HCC was alive after diagnosis and/or receiving resection as a study endpoint. ECOG performance status was evaluated since determination of large hepatic tumor, the scores would go up (worsen) over time mainly due to the tumor burden and cancer progression. For the resection group, the patients were referred to our clinic every 1 or 2 months to monitor the alpha-fetoprotein concentration and evaluate the tumor by computed tomography or magnetic resonance imaging. The preoperative and postoperative ECOG performance statuses were evaluated perioperatively; after the operation, the patients’ symptoms and complications of liver disease were treated accordingly. All patients received at least 6-month follow-up.

### Statistical analysis

The survival duration in the conservative group was calculated from the date of diagnosis to the date of death, and that in the resection group was calculated from the day of diagnosis to the date of death or last follow-up. Data was shown as mean ± standard deviation, median, or number. The assessed variables were age, sex, CCI, body mass index, ECOG performance status, and laboratory data including the serum alpha-fetoprotein level, platelet count, hemoglobin level, total bilirubin level, Child–Pugh score, tumor size, main portal vein thrombosis, and TNM staging. The chi-squared test and Student’s t-test were used to analyze the enumeration and measurement variables of the two groups, respectively. The cumulative survival rates were compared with the Kaplan–Meier method, and univariate and multivariate Cox proportional hazard analyses were performed to identify the factors associated with poor survival. Factors that emerged in the entire cohort with a *p* value of <0.20 were considered significant baseline covariates. They were adjusted by multivariate regression analyses with the forced entry method using SPSS 22.0 software (IBM Corp, Armonk, NY, USA) A *p* value of <0.05 was considered statistically significant.

## Results

**Figure 2:**
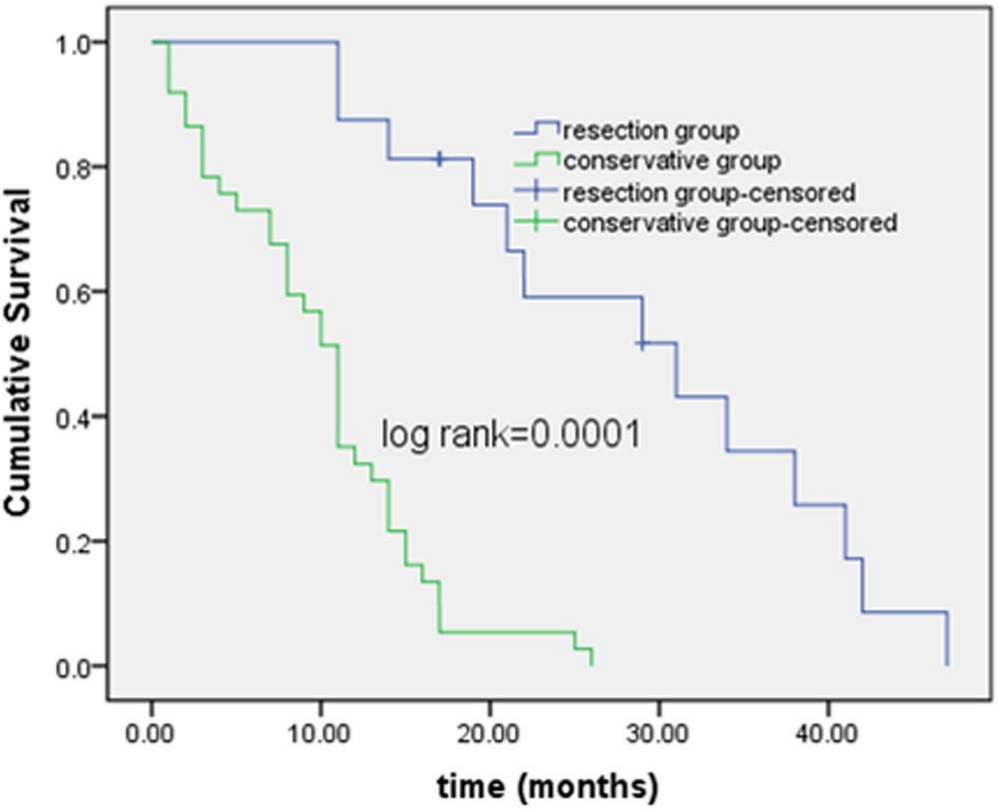
Survival of conservative group and resection one.

The resection group and the conservative one were compared in terms of their preoperational status, survival time, and ECOG performance status (Table 2). There were no differences in age, sex, liver function score, alpha-fetoprotein level, original diseases, hemoglobulin and platelets between the two groups. The median survival time of the conservative group and the resection one was 11 and 25.5 months, respectively (log-rank = 0.0001) (Fig. 2). The median ECOG performance status in the conservative and resection groups was 3 and 1 respectively (*p* = 0.023); the performance status worsened over time in the conservative group (from 2 at the time of diagnosis quickly to 5 at the end of life in most patients), CCI was different (*p* = 0.019). For the conservative group, 21 patients died in 1 year, two patients were alive for >24 months, and none alive for >3 years. The 3-month, 1-, 2-, and 3-year survival rates were 78.4%, 43.2%, 5.4%, 0% respectively. Of the deaths, 23 died of tumor progression, 11 of liver failure and 3 of cardiopulmonary disease.

In the resection group, hepatectomy was performed in 16 older patients with tumors of >10 cm. HCC in 16 patients were histologically diagnosed as primary hepatocellular carcinoma staged I to IIIb and well to moderately differentiated (Table 3). Eight patients developed wound liquefaction after operation, and all healed within 2 to 3 more weeks; one patient developed incisional hernia, which was surgically repaired with mesh 8 months later. One patient underwent a re-laparotomy for hemostasis 8 hours following hepatectomy. Portal vein thrombosis occurred in one patient and detected in routine re-examination one month after hepatectomy, no clinical presentation was observed and hence no intervention was applied. The rate of complications classified as Clavien–Dindo grade ≥IIIa was 12.5% (2/16). 11 patients died of cancer recurrence in 3 years after hepatectomy, two died of cardiac diseases and one died of unknown cause. The 3-month, 1, 2, 3-year survival rate was 93.7%, 56.3%, 12.5%. Recurrent cancer was detected in 7 patients in 12 months after hepatectomy, 5 more detected in 2 years and 3 more detected after 2 years, only one was alive with recurrence-free for over 2 years; the one, 2, 3 year cancer recurrence rate was 43.8%, 75.0% 93.8% respectively (Table 4). Multivariate Cox analysis showed the Charlson comorbidity index and the performance status associated with poor outcomes of those patients (*p* = 0.001, 0.018, respectively) (Table 4).

**Table 3:** Post-operational characteristics of patients in resection group.

No	Resection	Blood loss (ml)	Complications grading	Relapse (months)	ECOG score	Death reasons (months)
1	Sg6,7^+^	100	IIIb (hernia)	7	1	Relapse (47)
2	Sg2,3,4	800	no	6	0	Relapse (14)
3	Sg5,6,7	400	I (wound)	14	1	Relapse (31)
4	Sg5,6,7,8	1200	I (wound)	6	0	Relapse (27)
5	Sg6,7^+^	550	I (wound)	25	0	Alive (29)
6	Sg5,6,7,8	300	I (wound)	26	1	Cardiac (38)
7	Sg5,6,7,8	2600	II (Bleeding^#^)	no	0	unknown (34)
8	Sg5,6,7	750	IIIb^*^ (reopen)	13	0	Cardiac (34)
9	Sg 5,6,7^++^	965	I (wound)	29	0	Relapse (41)
10	Sg4,5,8	800	I (wound)	12	0	Alive (19)
11	Sg5,6,7,8	3100	PV thrombosis	5	1	Relapse (11)
12	Sg5,6,7	400	I (wound)	13	1	Relapse (21)
13	Sg2,3,4,5	400	II	9	2	Relapse (19)
14	Sg5,6,7	300	II	10	2	Relapse (19)
15	Sg4,5,6,7	700	I (wound)	13	1	Relapse (17)
16	Sg5,6,7	1400	no	17	1	Relapse (29)

^+^Represented enucleation of other small tumors; * represented relaparotomy for massive blood loss. Wound denoted wound heal delay due to liquefaction; bleeding^#^ for blood loss after closure of abdominal wall and wound liquefaction.

**Table 4:** Factors affecting survival of 2 groups.

Variables	B	*p*
Age	0.021	0.235
CCI	−0.489	0.001
Tbil	0.064	0.781
AFP*	0.16	0.689
ECOG	0.405	0.018

## Discussion

Reports of resection of large hepatic tumors in older patients are scarce in the literature. According to the Barcelona Clinic liver cancer staging system, large hepatic carcinomas are categorized as intermediate-stage tumors; these patients should receive only palliative care such as chemoembolization^16–19^. For older patients with large liver tumors, surgery is often preemptively denied at the discretion of surgeons who perceive the patients to have a limited remaining lifetime and will shoulder higher risks of major complications; these patients predominantly undergo conservative treatments such as TACE or cryosurgery.

Hepatectomy of large liver tumors has achieved satisfactory results worldwide and evolve to be the standard treatment modality for liver tumors as great progress was made in hepatic surgery, renovation of surgical instruments, and new insights into liver anatomy and pathophysiology^20–22^. In our daily practice, almost no patient is refused surgery or directly submitted to palliative supportive care only because of a potentially risky resection. In our report, HCC was staged as I-II in most cases, higher stages of HCC occurred in a fewer cases, they can be removed through partial hepatotectomy to achieve the longer lifespan, and occasionally some large liver tumors are benign or metastatic (data not shown). On the other hand the capability of liver regeneration and synthetics in older patients may be lower than that of the younger, older patients are capable of restoring their previous liver size^23^. The sufficient liver volume for a given patient is required to maintain support of the body after hepatectomy. No patients in the present study developed liver failure. Cardio-cerebral disease and diabetes mellitus were common comorbidities in our older patients, these conditions were successfully controlled with medications and had only small impacts on recovery^24^, and CCI was different between two groups due to cancer load and associated with the poor outcomes.

The favorable outcomes achieved in this study were underlined by surgical experiences and discreet assessment. Our study substantiated that hepatectomy is as effective for older patients as for other cohorts^25^; it achieved a prolonged life span and better the performance status which have a positive impact on them. The surgical complications such as wound liquefaction, incisional hernia, portal vein thrombosis were the same as in other cohorts undergoing hepatectomy; most were not age-related^26^. One concern regarding the surgical treatment of large liver tumors in patients of advanced age is the indications for such treatment; this concern is related to overtreatment. Actually, the natural history of liver cancer without surgery is poorly defined. Yeung *et al*. reported an overall median survival of 3 months, and their 1-year survival rate was only 7.8%^8,27^. Any patient desires to live longer and enjoy a higher quality of life. Patients with cancer, however, may be overwhelmed by their potentially incurable disease and in a state of distress; and their performance of daily activities may be compromised, depression, despair, or suicide may ensue, necessitating the surgical treatment for cancer. In our study, surgery definitely outperformed conservative treatments, even for patients with cancer recurrence, and the patients enjoyed a higher quality of life due to better performance statuses, which should not be marginalized due to surgeons’ presumption of a limited remaining lifetime. Surgeries should be considered in such cases to prolong the patients’ lifetime if possible. Additionally, some patients may be misdiagnosed preoperatively, large tumors may press on and inflate the adjacent tissues or organs; these patients may be subjected to prolonged distress without curative treatments. Thus, a nihilistic approach to hepatectomy for older patients with large liver tumors should be avoided. Further studies are needed to optimize the diagnosis and treatment based on the individual patient’s liver tumor scenario.

This was the first cohort study for over 70 year old patients with HCC larger than 10 cm who underwent hepatectomy and achieved the better prognosis compared with the conservative cares. Two factors contribute to desirable outcomes in older patients with large liver tumors. First, hepatectomy will likely eliminate the cancer burden, improve the ECOG performance status, and provide psychological relief. Second, the biological behavior of cancer in the older population differs from that in younger patients; tumors in older patients may be inactive and progress slowly. Third, most tumors were solitary in our report which were amenable to hepatectomy. In patients with cancer relapse, surgery would not eliminate but instead decrease the cancer load (eg, onco-reductive surgery); this treatment paves a pathway for subsequent palliative interventions such as TACE and cryosurgery.

This study also has some limitations. It was retrospective that conservative group served as the historical control in which selection bias was seemingly unavoidable. Furthermore, these patients were evaluated as suitable for resection group, and their general conditions were likely better than those of patients in the conservative group; this might have carried some selection bias and this may be due to small sample size although combination of large HCC and advance age is rare. Extensive studies involving multiple centers are needed to accumulate more cases and allow the development of a cost-effective strategy for resection of large liver tumors in older patients.

## Conclusions

Hepatectomy of large HCC remains the preferred treatment option for selected older patients, patients can enjoy a prolonged lifetime with great psychological relief, even with cancer load.
